# Dr. Roberton's Additional Remarks on Stricture

**Published:** 1809-08

**Authors:** 


### Dr. Roberton's additional Remarks on Stricture.

( Concluded from our last, pp. 17—28. )

In page 210, Case V, appears one of simple gonorrhœa, in which the increased contraction of inflamed parts caused by the belief of stricture, leads Mr. Home to a train of unnecessarily severe practice.

Case

CASE VII is one of Gleet.—Mr. Home for the most part informs us, that a gentleman from the *East Indies* applied to him with a case of stricture, &c. and this mode of statement doubtless precludes question; but sometimes he relates more distinctly the history of the case, and shows as if he suspected the presence of stricture on very slight grounds; in this case, for instance, the person had a gleety discharge, incontinence of urine, erections, and nocturnal emissions; in short, general depravation of health, succeeding gonorrhœa. Mr. Home supposes this to be a case of stricture, and not recollecting that irritation produces contraction, he, because his bougie was opposed by an obstacle which itself might have excited, was certain that there was stricture; consequently he destroyed all opposition by the caustic; and says, " I applied the caustic to this stricture three different times at the usual intervals, and the passage then admitted a common sized bougie. Finding that, in other cases, the passing a bougie, under these circumstances, brought on irritation, I did not propose the use of it, and left the parts entirely to themselves." Now, if he had observed the circumstances accurately, he would say, the irritation of the bougie and caustic brought on an inflammation of the urethra, which cured the gleet.

CASE VIII exhibits a spasmodic affection of the urethra, or rather the sphincter vesicæ, which was removed by a hemorrhagy which the caustic induced; *but might have been equally well, and for the patient, far more easily removed by leeches applied to the parts.*

In Case IX. a young man from the country, calls on Mr. Home to tell him that he was perfectly cured of a stricture; Mr. Home advises him never to travel without bougies, then introduces one into the canal; a contraction is perceived, Mr. H. *applies the caustic!* "The application," says he, " was repeated four times before the passage allowed a full sized bougie to go through the stricture; it was, however, much larger than any that had been passed before; I then desired that the parts might be left entirely to themselves, and *not* disturbed by passing a bougie; in this state the young man was at last suffered to return into the country." If Mr. Home had not disturbed the parts by his bougie, at a time when the patient had no complaint, would not the patient have been exempt from the idea of stricture, and all the painful treatment to which he was subjected?

CASE X. is one of gleet, considered as stricture, and cured by the caustic.

Case XI. is one of inflammation of the urethra and neighbouring parts.

These cases of stricture are of a singular nature; there is no impediment to the urinary evacuation, nor any want of retention; in other words, the constricted canal is as wide as usual.

Case XII. is one of gonorrhœa; first, properly treated and cured by a judicious surgeon. Though the gonorrhœa returned, proofs of stricture are deficient till after the application of bougies.

This is a case of mismanaged gonorrhœa running into gleet, and protracted four years.—One surgeon thought it a gonorrhœa, and removed the discharge by injection; another supposed it stricture, and also removed the discharge by bougies; Mr. Home at last produced a cure by means of caustic. This case, after the gonorrhœal inflammation had subsided, was converted into a bad gleet, and, as in other obstinate cases of this nature, the common remedies alleviated, but did not remove it, so that when any one introduced a bougie, the irritation made the canal contract round the instrument, and hence the idea of a stricture, which in reality only existed while the irritation of the bougie continued. At last, Mr. Home, under the idea of curing stricture, cauterized the canal, and, by this inflammation, removed the gleet; which could at first have been much better and far more easily accomplished in a few weeks.

In this whole section, the proofs of stricture are deficient in every case :—Case IV. it appears, baffled even the caustic to remove the discharge, and the patient remained uncured. This affection, if I may judge not only from my own experience, but from that I believe almost of many others now in the habit of treating these diseases, could have been completely cured by the internal use of cantharides.

In the cases of Section III. page 272, it is related, that a small tumour like a pea was distinctly felt in the urethra. What is this but the caruncle of Wiseman and others? Mr. H. supposes this tumour to arise from the thickened edge of a lacuna; but might not excrescence of this nature arise from any diseased surface? I am much inclined to believe, that in all the cases of this section, this pea-like body, or something similar, was the real cause of obstruction, and that the manifold strictures found in each canal were caused by the irritation of the bougies.

It appears that, in some instances, the stricture was rather ther the effect than the cause of fistula in perinæo, as in Case V. p. 312. "A gentleman had a stricture which was not known till it had produced a fistula in perinæo."

Mr. Home informs us frequently, that the hardness in perinæo is removed by the application of the caustic to the stricture, and the hardness is the only proof of the stricture existing. See Case V. p. 314, &c. But the tumour would have yielded much more readily, if the caustic had been applied in powder to its own surface externally, or, perhaps, even to the application of a blister over the external surface of the tumour. It is a fact, which I have often seen realized, that a piece of caustic applied, for instance, over the surface of a bubo for the purpose of opening it, a practice adopted by some, diminishes the size of it in one or two days, and by a repetition of the same practice, once or twice afterward, the tumour has entirely disappeared without bursting.

In Case VI. p. 316, a swelling between the fundament and scrotum had existed years before a stricture seemed to have formed. The case seems to have been a gleet, accompanied with occasional spasms aggravated by the bougies, and at last cured by the inflammation induced by the caustic. This disease had remained about nine years. Mr. Home concludes the case thus: "After the removal of the strictures in the urethra, a spasm came upon the bladder in the middle of the night, attended with suppression for several times, generally only every other night, and then went off." Was there not as much reason now to apply the caustic for stricture as formerly? We indeed often find that the same symptoms do not induce a repetition of the treatment; I must conclude that once was found enough, although it is not so expressed.

Page 322 is a Case of occasional spasm, arising from the stimulus of the urine or of the semen, on the tender urethra; and the last case in this section is a well marked case of inveterate gleet, with occasional spasm, cured by the caustic at last.

Case I. p. 338, entitled, "Stricture attended with complaints of the stomach and eruptions on the skin," does not seem to be any thing else than an inveterate gleet, accompanied as usual with general debility, and depravation of the appetite, &c. which affection was removed by the local application of caustic, and the internal use of corrosive sublimate!

Case IV. p. 345, entitled, "Stricture with nervous fever," affords insufficient proof that stricture existed independently pendently of the bougies; but the mucous discharge which is mentioned indistinctly, and the general debility, indicate something like gleet. Those who consult authors, will find that all the symptoms detailed by Mr. Home, viz. nervous affections, restlessness, quick small pulse, uneasy and disturbed sleep, with heat in the skin, and mucous discharge, nay, I may add, all the symptoms of hectic fever, are brought on by excess of venery, in any form where no strictures exist. In page 346, of the same case, "he had," says Mr. H. "no apparent difficulty in voiding urine, nor did he believe that the stream was smaller than is natural." Is the existence of a disease then only to be proved by its cure?

Case III. p. 355, is a case of Gleet.—Nor does Mr. H. afford us any proofs that the prostate gland was diseased.

Case I. p. 362, is pronounced stricture, though the symptoms appear the same as those in Case IV. p. 357, which is deemed not to be a stricture.

I cannot avoid quoting the following Case, p. 363. "A gentleman," says Mr. Home, "aged 30, had a frequency in making water, particularly in the forenoon, which continued through the day, but went off entirely on going to bed, and he did not make water till he got up in the morning. He had also a gleet, as it was termed, in consequence of gonorrhœa, which had continued upon him for two years.—From the frequency in making water and the discharge! I was naturally led to suspect there was a stricture, and therefore examined the urethra by passing a bougie. I met with a stricture at five inches; this was removed by the caustic; another was found at six inches and a half, which was also destroyed, and the bougie passed with ease into the bladder. The parts were now left to themselves, and the symptoms continued without any abatement. At the end of a month, the bougie was passed to ascertain whether the stricture had been entirely removed, and it passed with great ease. The circumstance of the bladder being at ease during the whole night made me suspect stone, which by its motion gave uneasiness, but none when at rest. I sounded the bladder, but nothing hard was felt. The disease appears therefore to be an irritated state of the membrane of the bladder, probably brought on by the stricture.—In this case there was little sediment in the urine. By the use of the mephitic alkaline water, the patient has almost entirely got the better of his complaints."

This

This is a simple case of gleet brought on by gonorrhœal inflammation, but Mr. Home says, "the disease appears to be an irritated state of the membrane of the bladder, probably brought on by the stricture." The caustic, however, did not cure this patient; for Mr. Home concludes the case thus, "this patient has *almost entirely* got the better of his complaints."

Case I. p. 373, is a very well related and ably treated case of stricture and stone in the urethra. This case proves that an irritating substance applied to the urethra, makes it contract so as to form an impediment to the passing of the substance.

Case II. p. 435, which Mr. Home gives as an instance of hydrcoele cured by the removal of stricture, was probably a case of gleet combined with hydrocele. The caustic was applied to the urethra, which removed the gleet by inducing inflammation; and, at the same time, the hydrocele was cured by stimulating the vessels of the neighbourhood.

Mr. Home, in page 442, says, that the swelling of the perinæum in consequence of caustic, is entirely produced by the blood extravasated in the cellular membrane; but of this we have no proof, and the facts related seem to me to indicate otherwise.

Case II. p. 444, was gleet succeeding gonorrhœa; in which the injudicious application of the caustic produced very bad symptoms, without producing a cure.

Case I. p. 474, seems only an occasional spasm.

Case II. p. 475, very completely proves that the irritating substances, as bougies and the caustic, may produce very dangerous constrictions in the urethra.

The Cases adduced, p. 484, &c. in support of stricture being the cause of ague, and which disappeared when these were removed, are by no means sufficiently supported. By a careful examination in the early stages of them, it will appear probable, that they were spasmodic stricture; that the patients resided in parts of the world where these diseases are common, and in all probability in the neighbourhood of places which gave origin to such complaints; that on the return of the patients, as has been formerly stated, to this country, where no such causes operated, they would soon have recovered without the bougies. They were probably spasmodic strictures, occasioned partly, perhaps, from the effect of the bougie, but more likely from the horror into which the patient was put at the very thought of such a barbarous operation.

The Chapter, p. 494, on the treatment of strictures in the the œsophagus, is certainly a very extraordinary one. If, as I suspect, the disease arose from hysteria, combined with affections of the digestive organs, such severe treatment must have been unnecessary.

As a very great degree of similarity may be found in Mr. Home's reasoning in both volumes, to be even as minute in my examination of the second as I have been of the first, generally as I have treated the subject, might lead to unnecessary repetition; I shall therefore take notice of but very few of Mr. Home's cases, enough having been said on that part of the subject.

Any person who may have read Mr. Home's first, and as far as page 46 of his second volume, may feel surprised, after all his reasoning, and the numerous cautions held out to others, that even he should have fallen into such an error, as he is candid enough to acknowledge in that section. The case he has adduced in proof of irritation in the urethra from inflammation in the internal membrane of the bladder, is simply a spasmodic affection. The disease, Mr. Home informs us, was mistaken by a surgeon in London for stricture, to which he applied caustic, and thus, by destroying every obstruction, gave momentary relief, but did not in the least remove the tendency to contraction. Thus continually harrassed, and having suffered greatly from hemorrhage, the life of the patient was brought into the utmost danger. In this state he applied to Mr. Home for advice, who was, *by advice of his patient*, prevailed on to employ the caustic, although it had been so frequently used before, without yielding any thing more than temporary relief, and although he himself gives it as a case of no disease in the urethra, but one of irritation from inflammation of the bladder. The patient at length died, and the following were the appearances on dissection.

Mr. Home informs us, that "upon inspecting the parts after death, it appeared that there had been no stricture in any part of the urethra. The internal membrane of the bladder was in a state of ulceration, particularly the lower part, where the ureters enter into it, except a line not broader than one-eighth of an inch, extending from each ureter to the middle line, where the two streams would unite. The orifices of the ureters were in a state of ulceration, and inflammation had extended itself all along the internal surface of the left ureter to the kidney, the pelvis and infundibula of which were in a state of ulceration."

"The use of the caustic, he adds, had made *five different*

*ferent holes* through the membrane of the urethra, of the size of the end of the common bougie, at a small distance from each other ; and *a large abscess had formed between the perinæum and buttock, into which the urine escaped by these orifices.*" Good God! what could have tempted Mr. H. to make such a case known to the world? Why has he voluntarily exhibited such a mass of blunders and cruelties committed, not only by another surgeon, but by himself? Why, if another surgeon went wrong, did he persevere in a similar plan of practice? What became of all his advice and all his reasoning on such an occasion? And above all, when he did commit the fault, why did he expose himself by exhibiting the urethra with five holes in the various parts to which the caustic had been applied by mistake; with a large abscess formed in the perinæum from the inflammatory action, and the internal membrane, &c. of the bladder in a state of ulceration, probably from the same rude treatment!

It seems from the history of this case, that, even from the very commencement, by the properly regulated use of internal medicines and external applications, this patient might have recovered.

I again urge, that the extreme difficulty of applying the caustic immediately to the strictured part, independently of every other consideration, is very great, and, perhaps, there are few surgeons capable of hitting the mark. The consequences of such an error must then be extremely frequent; even by Mr. Home's own confession, in some of his cases it has occurred to him, and, conscious of the danger, he makes the following observation in page 57, "To accomplish this, requires great attention on the part of the surgeon; since the smallest inaccuracy in the application of the caustic, occasions it to get beyond the natural boundaries of the urethra, and the smallest excess of violence brings on too much inflammation, and consequently, in such thickened parts, a suppression of urine; while, on the other hand, too much mildness prevents the patient from making any advance towards recovery."

These circumstances must render burning with the caustic an operation of a most ticklish nature, which ought never to be attempted till every other rational method we can devise has completely failed of success, and even then only by people who know what they are doing.

Case III. p. 127, is advanced as a proof of the *effect of stricture on the bladder*; Mr. Home introduces it thus, "The following case I am particularly anxious to lay before fore the public for several reasons; it was one which Daran and every surgeon since his time had taken charge of without success. It is one in which Mr. Hunter tried the caustic without performing a cure, &c." This Case is detailed to the length of 100 pages, and we find that Mr. Home was equally unsuccessful. It appears that this was a disease of the bladder itself and left kidney, and of the prostate gland, under which the patient had laboured 53 years; and on dissection we find evidence, that the affection of the bladder, &c. had extended itself to the urethra, which shewed no morbid symptoms, except at the very points where the caustic had been applied.

Although, undoubtedly, various substances existing in the kidnies, ureters, or bladder, occasion great irritation, and often violent contraction of the urethra, even when no disease exists exclusively in that canal, yet these contractions are almost always of a spasmodic nature, often originating in the urethra itself, in consequence of diseases of that canal; and by the harsh treatment of the surgeon, or long continuance of the disease, sometimes extend their influence to the bladder, ureters, or even to the kidnies.

On inspecting the body of the patient just alluded to, after death, the following appearances were observed.

"The urethra had one uniform smooth surface throughout its whole extent; there was no appearance of contraction in any part of the canal; but upon a minute examination, the spots, where the stricture at five inches, and that at seven inches had been, could be distinguished by the membrane being thin, (*of course by the burning with the caustic*) and more compact at these parts than in any other. The prostate gland was enlarged, and several different abscess had formed in its substance; these had opened into the cavity of the bladder, and the inflammation they had produced, had extended itself over the internal membrane, which was crusted over with coagulable lymph. This adventitious substance projected every where by very irregular processes into the cavity, and portions of it had, during life, been occasionally separated and voided with the urine." Nor can I doubt that the too liberal use of the caustic promoted the inflammation, aggravated the symptoms, and hastened the death of the patient.

In page 243, Mr. Home gives two Cases as instances *of stricture*, brought on by onanism, in neither of which is there proof that stricture existed previously to the introduction of the bougies. The first case is related in the following manner, p. 247. "A gentleman, who had early addicted addicted himself to that pernicious vice, had the following symptoms brought on at the age of 21; frequent emissions in sleep, attended with lassitude, depression of spirits, and loss of general health; head-ach; inability to apply his mind to business or exert himself, for the whole of the day after such an effect had taken place. These occasionally happened for several nights in succession, and then left him for six or seven, but that was the longest interval. The effect of these attacks upon his reasoning faculties was such, as to make him completely miserable. I explained to him, that I thought it probable, the symptoms of which he complained, arose from a spasmodic stricture *immediately behind the bulb of the urethra.*" What is there in these symptoms from which we should infer the presence of stricture? Not the general debility, lassitude, and depression of mind; for what could produce these more effectually than the inordinate exertion of these organs, every act of which, in the most natural way, is succeeded by that very state of mind and body! Not the nocturnal emissions, for such practices induce these quite independently of stricture; I have seen hundreds of such instances. Besides, the very diagnostic symptom is wanting, by which we have a right to infer the presence of stricture of any kind, viz. the diminished stream of the urine; but Mr. Home not only judges that there is stricture, but predetermines the very site of it, "immediately behind the bulb of the urethra." Now, as it appears by Mr. Home himself, that the urethra was "in an irritable state, and preternatural sensibility," we can easily perceive why it was contracted when the bougie was forced into it, and we are surprised from other relations by Mr. Home, that he did not suspect it in this case. The complaint of this patient, Mr. Home tells us, was very much relieved by the caustic.

In page 269, we find strictures producing other diseases. Section I, is entitled Erysipelas, in consequence of stricture; and on examining the case adduced in evidence of this, it seems probable that it was the bougies introduced into the urethra, and not the stricture, which caused erysipelas.

Section II, p. 271, is entitled, Sciatica, in consequence of stricture; and in the next page Mr. Home tells us, "The application of the caustic, and inflammation from other causes, produce the sciatica, but it does not appear to be an immediate symptom of stricture."

In all Mr. Home's speculations respecting permanent strictures, none is carried so far, as in the notions he has entertained of stricture in the œsophagus. There are few men men who do not, at some period or other of their lives, entertain very strange notions respecting many points of science; and it is not uncommon for men to publish their opinions on these subjects, even under the influence of these mistakes. But, in general, when they afterwards reflect seriously, it is more honourable for them to retract, than pertinaciously to insist on such notions being actually true.

We find, then, in Mr. Home's second volume, he still endeavours to support the notion of strictures in the œsophagus being by no means an uncommon disease: he assures us, in page 397, that strictures in the œsophagus are most common in the earlier periods of life, or, he might have added, when the passions are most strong, and when hysteria is most common. Mr. Home's strictures in the œsophagus almost all occurred in females, or in men in general of a delicate habit and irritable mind. In other words, they are commonly to be found in such persons as are most liable to violent hysteric affections.

The task I have now executed, has occasioned me many an unpleasant sensation. I waited with the utmost impatience for the publication of every book that was announced on the subject of stricture, in full confidence that some of those authors would anticipate what I had to say. I need scarcely state that I have been disappointed, for these works are now in the hands of the public, and may be consulted. Some of them, indeed, have started very strong objections to the use of the caustic bougie, but none of them have proposed any rational plan by which such obstructions could be removed, without, in many instances, occasioning more torture to the patient than human nature was capable of supporting.

It is extremely painful to make innovations on any generally received plan of practice; but I could not witness the severe methods, which on every hand presented themselves to me, without appealing to the public tribunal to avert a repetition of such acts.

---

*** We have inserted these long remarks on a practice which at present excites general attention; and shall thankfully receive any reply from our various Correspondents. We cannot conclude without observing, that whether all the cases relieved by caustic were really stricture or not, still the practice was successful after the usual remedies had failed. We are also obliged to express our surprize that stricture of the œsophagus should be so rare a disease in the northern metropolis.

*Account*

